# Healthy lifestyles in relation to cardiometabolic diseases among schoolteachers: A cross‐sectional study

**DOI:** 10.1002/hcs2.59

**Published:** 2023-07-10

**Authors:** Liyan Huang, Mengjie He, Jie Shen, Yiying Gong, Hui Chen, Xiaolin Xu, Geng Zong, Yan Zheng, Chao Jiang, Baohong Wang, Ronghua Zhang, Changzheng Yuan

**Affiliations:** ^1^ School of Public Health, The Second Affiliated Hospital Zhejiang University School of Medicine Hangzhou China; ^2^ Institute of Nutrition and Food Safety Zhejiang Provincial Center for Disease Control and Prevention Hangzhou China; ^3^ The Key Laboratory of Nutrition, Metabolism and Food Safety, Shanghai Institute of Nutrition and Health, University of Chinese Academy of Sciences Chinese Academy of Sciences Shanghai China; ^4^ Human Phenome Institute, School of Life Sciences Fudan University Shanghai China; ^5^ Zhejiang Provincial Key Laboratory of Cancer Molecular Cell Biology, Life Sciences Institute Zhejiang University Hangzhou China; ^6^ State Key Laboratory for Diagnosis and Treatment of Infectious Diseases, National Clinical Research Center for Infectious Diseases, Collaborative Innovation Center for Diagnosis and Treatment of Infectious Diseases, The First Affiliated Hospital Zhejiang University School of Medicine Hangzhou China; ^7^ Department of Nutrition Harvard T.H. Chan School of Public Health Boston USA

**Keywords:** healthy lifestyle, cardiometabolic diseases, schoolteachers, cross‐sectional study

## Abstract

**Background:**

We aimed to explore the associations of adherence to an overall healthy lifestyle with cardiometabolic diseases (CMDs) among schoolteachers in China.

**Methods:**

We conducted a cross‐sectional analysis among 2983 teachers (aged 39.8 ± 9.3 years, 73.8% women) in Zhejiang Province, China. A healthy lifestyle score (0–7) was constructed based on seven low‐risk factors: healthy diet, noncurrent smoking, noncurrent drinking, regular exercise, normal body mass index (BMI), adequate sleep duration, and limited sedentary behavior. CMDs included self‐reported hyperlipidemia, hypertension, diabetes, coronary heart disease, and stroke. Multivariable‐adjusted logistic regression models were used to evaluate the associations between healthy lifestyle and CMD.

**Results:**

A total of 493 (16.5%) participants had at least one CMD, with hyperlipidemia, hypertension, and diabetes being the three leading CMDs. Each point increment in a healthy lifestyle score was associated with 20% lower odds of having CMD (*p*‐trend < 0.001). Compared with 0–3 low‐risk factors, the odds ratios (*OR*s) and 95% confidence intervals (*CI*s) were 0.66 (0.50–0.88) for 4 low‐risk factors and 0.51 (0.39–0.67) for 5–7 low‐risk factors. We observed independent associations for normal BMI (*OR* = 0.50, 95% *CI* = 0.40–0.63), noncurrent drinking (*OR* = 0.53, 95% *CI* = 0.36–0.77), and limited sedentary behavior (*OR* = 0.77, 95% *CI* = 0.62–0.96) in relation to CMD. Healthy diet (*OR* = 0.75, 95% *CI* = 0.55–1.01) exhibited marginally significant association with CMD.

**Conclusions:**

Our findings suggest that adherence to an overall healthy lifestyle is associated with lower odds of CMD among schoolteachers.

## Graphical abstract

An overall healthy lifestyle (including healthy diet, noncurrent smoking, noncurrent drinking, regular exercise, normal body mass index (BMI), adequate sleep duration, and limited sedentary behavior) was significantly associated with lower odds of cardiometabolic diseases. Among specific lifestyle factors, normal BMI, noncurrent drinking, and limited sedentary behavior were the major contributors to the observed associations.

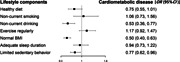

**Abbreviations**
AIC: Akaike Information Criterion
BIC: Bayesian Information Criterion
BMI: body mass index
CESD‐10: 10‐item Center for Epidemiological Studies Depression Scale
CHEI: Chinese Healthy Eating Index
CI: confidence interval
CMD: cardiometabolic disease
CVD: cardiovascular disease
DASH: Dietary Approaches to Stop Hypertension
GAD‐7: Generalized Anxiety Disorder 7‐Item Scale
OR: odds ratio
TAP: Teachers Ageing Project

## 1 INTRODUCTION

Cardiovascular disease (CVD), diabetes mellitus, and related conditions are considered as cardiometabolic diseases (CMDs) and are major causes of morbidity, disability, and impaired quality of life [1]. Approximately one in three adults worldwide was afflicted with CMD [2], making it a global healthcare priority.

A healthy lifestyle has been associated with lower risks of CMDs [3, 4, 5]. A recent prospective cohort study among 0.3 million participants in seven European countries found that adherence to a healthy lifestyle consisting of five factors—normal body mass index (BMI), noncurrent smoking, limited alcohol consumption, regular physical activity, and healthy diet—was associated with a 16% and 18% lower risk of CVD and type 2 diabetes, respectively, for each 3‐unit increment of healthy lifestyle score [1]. Another large‐scale study among 0.5 million Chinese adults reported similar associations of healthy lifestyles with the risk of a transition from healthy status to CMD [6].

However, evidence regarding the associations of healthy lifestyle with CMD among primary and middle school teachers is insufficient. Diverse lifestyle patterns might also exist between schoolteachers and the general population. It is also unclear whether and to what extent an overall healthy lifestyle is associated with CMD among teachers, who are under a lot of work‐related stress and potentially at risk of developing CMD. To fill this knowledge gap and provide scientific evidence for disease prevention and policy implementation among schoolteachers according to the “Healthy China Initiative 2019‐2030,” we examined the association of adherence to an overall healthy lifestyle (including a healthy diet, noncurrent smoking, noncurrent drinking, regular exercise, normal BMI, adequate sleep duration, and limited sedentary behavior) with CMD among schoolteachers in China.

## 2 METHODS

### 2.1 Study population and design

The Teachers Ageing Project (TAP) is an ongoing population‐based study initiated in 2022 among schoolteachers living in Zhejiang Province, China. Using a multistage convenience cluster sampling method, we enrolled 3223 teachers aged 22–66 years from 39 public and private schools (23 primary schools and 16 junior high schools) in six geographically and economically diverse cities (Hangzhou, Ningbo, Taizhou, Jiaxing, Lishui, and Quzhou) in Zhejiang Province. All included teachers completed a baseline web‐based questionnaire comprising demographic information, socioeconomic status, lifestyle factors, and health conditions. Teachers aged 50 years and above received a telephone‐based cognitive function assessment. Individuals with complete baseline surveys are to be followed up biennially. The overall goal of the TAP is to understand the health status of schoolteachers and to investigate the potential risk factors and health determinants, with a primary focus on nutritional factors of age‐related cognitive function. The medical ethics committee of the School of Public Health, Zhejiang University approved the study protocol (ZJL202203‐4), and all participants provided electronic informed consent. We excluded 240 participants without information on BMI and analyzed the data of 2983 participants.

### 2.2 Assessment of healthy lifestyles

We constructed a healthy lifestyle score based on seven behavioral factors according to related literature and guidelines, including diet, smoking, drinking, physical activity, BMI, sleep duration, and sedentary behavior [1, 7, 8]. Information on dietary intake was assessed using a food frequency questionnaire (FFQ) modified from a validated FFQ [9]. We assessed diet quality using the Dietary Approaches to Stop Hypertension (DASH) diet score, which has been shown to be related to cardiometabolic health [10]. The DASH score includes eight food groups: fruits, vegetables, nuts and legumes, dairy, whole grains, sodium, sugar‐sweetened beverages, and red and processed meats (Supporting Information: eTable 1). Greater adherence to the DASH diet (the highest quintile) was defined as a low‐risk factor. Smoking and drinking status were self‐reported as current, former, or never; we defined noncurrent smoking and drinking as low‐risk factors. Regular exercise was defined as engaging in at least 30 min of moderate and vigorous activity three or more times a week, based on the recommendations of the Healthy China Initiative 2019–2030. This definition was in line with the guidelines of the World Health Organization [11], which was based on the evidence summarized in reviews regarding the overall health (including cardiovascular health) benefit of physical activity [12]. BMI was calculated as self‐reported weight in kilograms divided by self‐reported height in meters squared, and normal BMI (18.5–24.0 kg/m^2^) was defined as a low‐risk factor [13]. We defined adequate sleep duration (7–9 h/day) [8] and limited sedentary behavior (less than 4 h/day) [7] as low‐risk factors. The healthy lifestyle score was obtained by counting the number of low‐risk factors and ranged from 0 to 7. Details of all definitions can be found in Supporting Information: eTable 2.

### 2.3 Assessment of CMDs

History of CMD was assessed according to a self‐reported diagnosis of diabetes, hypertension, coronary heart disease, stroke, and hyperlipidemia. The primary outcome of interest was any self‐reported CMD, and secondary outcomes included three most common CMDs: hyperlipidemia, hypertension, and diabetes. Moreover, health examination reports were collected in a subgroup of study participants. To verify the validity of self‐reported measures, we calculated the Spearman correlation coefficient between BMI based on measured data and self‐reported data, and the Kappa values of CMDs between cardiometabolic biomarkers and the corresponding self‐reported health status. Participants were defined as having type 2 diabetes (fasting plasma glucose ≥7.0 mmol/L, or glycosylated hemoglobin ≥6.5%) according to guidelines for the prevention and treatment of type 2 diabetes mellitus in China (2020) [14]. Hypertension (systolic blood pressure ≥140 mmHg or diastolic blood pressure ≥90 mmHg) was determined according to the national guideline for hypertension management in China (2019) [15]. Hyperlipidemia (serum total cholesterol ≥5.2 mmol/L or triglycerides ≥1.70 mmol/L) was assessed according to the Chinese guidelines on the prevention and treatment of dyslipidemia in adults (2016) [16].

### 2.4 Assessment of covariates

We included multiple sociodemographic and health‐related factors in the adjustment of confounders. The sociodemographic characteristics included age, sex, education level, income level, current residential area, birthplace, and coresident status. A family history of CMDs was defined according to self‐reported information about whether the first‐degree relatives of participants had any chronic conditions including diabetes, hypertension, myocardial infarction, and stroke. Major mental disorders were assessed using two validated scales, the 10‐item Center for Epidemiological Studies Depression Scale (CESD‐10) and Generalized Anxiety Disorder 7‐item Scale (GAD‐7). Participants with CESD‐10 scores ≥12 (range, 0–30) were defined as having depressive symptoms [17], and those with GAD‐7 scores ≥10 (range, 0–21) were defined as having anxiety symptoms [18].

### 2.5 Statistical analysis

The baseline characteristics of study participants are presented as mean and standard deviation for continuous variables and number (percentage) for categorical variables. For group comparisons, one‐way analysis of variance was used for continuous variables, and the chi‐square test was used for categorical variables.

To evaluate the associations between healthy lifestyle scores and CMD, we used logistic regression models to estimate the odds ratios (ORs) and 95% confidence intervals (*CI*s). Associations with CMD of categorical healthy lifestyle scores (in tertiles) and the continuous score (each point increment) were modeled separately. Model 1 was adjusted for age (<30, 30–34, 35–39, 40–44, 45–49, ≥50 years) and sex. Model 2 was additionally adjusted for marital status (married, other), education level (high school and below, bachelor's degree, master's or doctorate), personal income level (in tertiles), residence (rural, urban), birthplace (rural, urban), and living alone (yes, no). In model 3, we additionally adjusted for a family history of CMD (hypertension, diabetes, myocardial infarction, and stroke). In the secondary analysis, we examined the association between individual low‐risk factors and CMD, and estimates of the seven factors were mutually adjusted. Spearman correlation coefficients were calculated among lifestyle factors and other major covariates. Multicollinearity tests were also conducted to assess the possibility of multicollinearity. We also examined the associations of a healthy lifestyle with hyperlipidemia, hypertension, and diabetes. Stratified analysis was conducted to explore the associations across subgroups defined by the main covariates, including age, sex, marital status, income, residential area, birthplace, and co‐resident status. *P*‐interactions were calculated by including a cross‐product interaction term in the multivariable‐adjusted models.

To test the robustness of the associations, several sensitivity analyses were performed. First, we further adjusted the models for depressive and anxiety symptoms. We also redefined a healthy diet as greater adherence to the Chinese Healthy Eating Index (CHEI) and repeated the primary analysis. Additionally, we redefined the normal sleep duration as 6–8 h/day [7]. Moreover, a modified healthy lifestyle score was summarized using five modifiable lifestyle factors, including diet, smoking, drinking, physical activity, and BMI, to examine the associations between a healthy lifestyle and CMD. Finally, we used generalized linear mixed‐effect models with the school as the random effect to control the potential cluster effect. The Akaike information criterion (AIC), Bayesian information criterion (BIC), and *R* ^2^ were reported to depict the power of the models. Analyses were conducted using R version 4.1.2 (The R Project for Statistical Computing, Vienna, Austria). Two‐sided *p* < 0.05 was considered statistically significant in all analyses.

## 3 RESULTS

Among 2983 study participants, the mean age was 39.8 years, 2201 (73.8%) were women, and 493 (16.5%) had CMD (Table 1). The number of participants with hyperlipidemia, hypertension, diabetes, coronary heart disease, and stroke was 341 (11.4%), 204 (6.8%), 71 (2.4%), 18 (0.6%), and 7 (0.2%), respectively. Supporting Information: eTable 3 shows the age‐ and sex‐specific distributions of each lifestyle factor. Compared with participants in the lowest tertile of healthy lifestyle scores, those in the highest tertile were more likely to be women, married, born in urban areas, have higher income, live in urban areas, and were less likely to live alone or to have depressive symptoms and anxiety symptoms. The Spearman correlation coefficient of self‐reported and measured BMI was 0.95 (*n* = 46). The Kappa coefficients between cardiometabolic biomarkers and self‐reported health status were 97.7% for type 2 diabetes (*n* = 88), 94% for hypertension (*n* = 50), and 67.8% for hyperlipidemia (*n* = 59) (Supporting Information: eTable 4). The correlation matrix for the main covariates is shown in Supporting Information: eTable 5, with all Spearman correlation coefficients <0.5. Multicollinearity tests showed a low likelihood of multicollinearity (all variance inflation factors <2; data not shown).

**Table 1:** Baseline characteristics of participants.

Variables	Overall	Healthy lifestyle score			*p* value
		Low (0‐3 low‐risk factors)	Medium (4 low‐risk factors)	High (5‐7 low‐risk factors)	
*N*	2983	657	980	1346	
Age, mean ± SD, years, *n* (%)	39.8 (9.3)	39.9 (9.9)	38.7 (9.2)	40.5 (9.1)	<0.001
<30	563 (18.9)	141 (21.5)	209 (21.3)	213 (15.8)	<0.001
30–39	861 (28.9)	164 (25.0)	318 (32.4)	379 (28.2)	
40–49	1053 (35.3)	229 (34.9)	317 (32.3)	507 (37.7)	
≥50	506 (17.0)	123 (18.7)	136 (13.9)	247 (18.4)	
Gender, *n* (%)					<0.001
Male	782 (26.2)	283 (43.1)	262 (26.7)	237 (17.6)	
Female	2201 (73.8)	374 (56.9)	718 (73.3)	1109 (82.4)	
Marriage, *n* (%)					<0.001
Married	2382 (79.9)	490 (74.6)	769 (78.5)	1123 (83.4)	
Other	601 (20.1)	167 (25.4)	211 (21.5)	223 (16.6)	
Education level, *n* (%)					0.773
High school and below	24 (0.8)	5 (0.8)	6 (0.6)	13 (1.0)	
College	2798 (93.8)	621 (94.5)	921 (94.0)	1256 (93.3)	
Above	161 (5.4)	31 (4.7)	53 (5.4)	77 (5.7)	
Personal income level, *n* (%)					0.016
Low	533 (17.9)	123 (18.7)	202 (20.6)	208 (15.5)	
Medium	2271 (76.1)	501 (76.3)	721 (73.6)	1049 (77.9)	
High	179 (6.0)	33 (5.0)	57 (5.8)	89 (6.6)	
Living alone, *n* (%)					0.019
Yes	258 (8.6)	67 (10.2)	96 (9.8)	95 (7.1)	
No	2725 (91.4)	590 (89.8)	884 (90.2)	1251 (92.9)	
Residence place, *n* (%)					<0.001
Rural	440 (14.8)	124 (18.9)	153 (15.6)	163 (12.1)	
Urban	2543 (85.2)	533 (81.1)	827 (84.4)	1183 (87.9)	
Birth place, *n* (%)					0.041
Rural	2094 (70.2)	478 (72.8)	702 (71.6)	914 (67.9)	
Urban	889 (29.8)	179 (27.2)	278 (28.4)	432 (32.1)	
Healthy diet, *n* (%)	567 (20.5)	20 (3.3)	71 (7.8)	476 (38.0)	<0.001
Noncurrent smoking, *n* (%)	2701 (90.5)	502 (76.4)	896 (91.4)	1303 (96.8)	<0.001
Noncurrent drinking, *n* (%)	2488 (83.4)	456 (69.4)	836 (85.3)	1196 (88.9)	<0.001
Exercise regularly, *n* (%)	1135 (38.0)	100 (15.2)	218 (22.2)	817 (60.7)	<0.001
Normal BMI, *n* (%)	1989 (66.7)	196 (29.8)	634 (64.7)	1159 (86.1)	<0.001
Normal sleep duration, *n* (%)	2313 (77.5)	296 (45.1)	771 (78.7)	1246 (92.6)	<0.001
Limited sedentary behavior, *n* (%)	1482 (49.7)	97 (14.8)	367 (37.4)	1018 (75.6)	<0.001
History of CMD, yes, *n* (%)	493 (16.5)	166 (25.3)	152 (15.5)	175 (13.0)	<0.001
Diabetes	71 (2.4)	20 (3.0)	21 (2.1)	30 (2.2)	0.446
Hypertension	204 (6.8)	75 (11.4)	53 (5.4)	76 (5.6)	<0.001
Coronary heart disease	18 (0.6)	7 (1.1)	5 (0.5)	6 (0.4)	0.219
Stroke	7 (0.2)	5 (0.8)	1 (0.1)	1 (0.1)	0.007
Hyperlipidemia	341 (11.4)	123 (18.7)	114 (11.6)	104 (7.7)	<0.001
Family history of CMD, yes, *n* (%)	1518 (50.9)	352 (53.6)	500 (51.0)	666 (49.5)	0.226
Diabetes	532 (17.8)	118 (18.0)	178 (18.2)	236 (17.5)	0.922
Myocardial infarction	54 (1.8)	12 (1.8)	19 (1.9)	23 (1.7)	0.918
Hypertension	1377 (46.2)	328 (49.9)	463 (47.2)	586 (43.5)	0.019
Stroke	210 (7.0)	65 (9.9)	63 (6.4)	82 (6.1)	0.005
Depressive symptoms, *n* (%)					<0.001
Yes	689 (23.1)	205 (31.2)	231 (23.6)	253 (18.8)	
No	2294 (76.9)	452 (68.8)	749 (76.4)	1093 (81.2)	
Anxiety symptoms, *n* (%)					<0.001
Yes	240 (8.0)	87 (13.2)	81 (8.3)	72 (5.3)	
No	2743 (92.0)	570 (86.8)	899 (91.7)	1274 (94.7)	

Abbreviations: BMI, body mass index; CMD, cardiometabolic disease.

**Figure 1:**
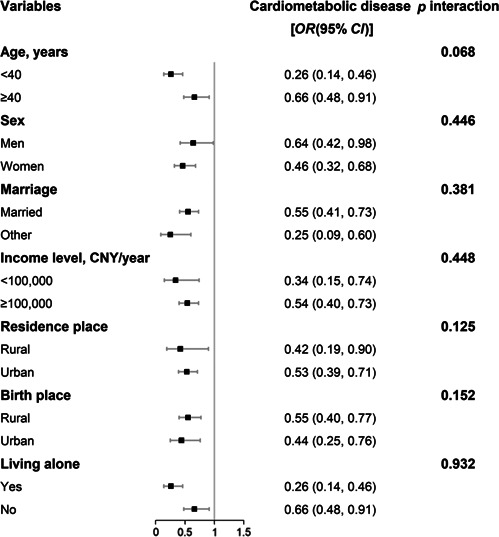
Subgroup analysis for the association between healthy lifestyle score and cardiometabolic diseases. Models were adjusted for age, sex, marital status (married, other), education level (high school and below, college, above), income (low, medium, high), residence (rural, urban), birthplace (rural, urban), living alone (yes, no), and family history of cardiometabolic diseases (any of hypertension, diabetes, myocardial infarction, and stroke).

After adjustment for sociodemographic characteristics and family history of CMD, higher healthy lifestyle scores were significantly associated with lower odds of CMD. Compared with 0–3 low‐risk factors, the multivariable‐adjusted ORs (95% *CI*s) were 0.66 (0.50–0.88) for 4 low‐risk factors and 0.51 (0.39–0.67) for 5–7 low‐risk factors. In particular, each unit increment in healthy lifestyle score was associated with 20% (95% *CI* = 12%–27%) lower odds of having CMD (*p*‐trend < 0.001) (Table 2). The performance of the models is shown in Supporting Information: eTable 6. The AIC, BIC, and *R* ^2^ for the final adjusted model were 2199, 2294, and 19%, respectively. Moreover, similar associations were observed across major study subgroups by age, sex, marital status, income, residential area, birthplace, and co‐resident status (Figure 1).

**Table 2:** Association between healthy lifestyle scores and cardiometabolic diseases.

	Health lifestyle score			Continuous (per 1 unit increment)	
Models	Low	Medium	High		*p* trend
Score range	0–3	4	5–7	0–7	
*N*	657	980	1346	2983	
Model 1	Ref (1.00)	0.66 (0.50, 0.87)	0.49 (0.37, 0.64)	0.79 (0.72, 0.86)	<0.001
Model 2	Ref (1.00)	0.66 (0.50, 0.86)	0.49 (0.37, 0.64)	0.79 (0.72, 0.86)	<0.001
Model 3	Ref (1.00)	0.66 (0.50, 0.88)	0.51 (0.39, 0.67)	0.80 (0.73, 0.88)	<0.001

*Note*: Model 1 adjusted for age (<30, 30–39, 40–49, ≥50 years) and sex.

Model 2 additionally adjusted for marital status (married, other), education level (high school and below, college, above), income (low, medium, high), residence (rural, urban), birthplace (rural, urban), and living alone (yes, no).

Model 3 additionally adjusted for family history of cardiometabolic diseases (any hypertension, diabetes, myocardial infarction, and stroke).

Abbreviation: Ref, Reference group.

**Figure 2:**
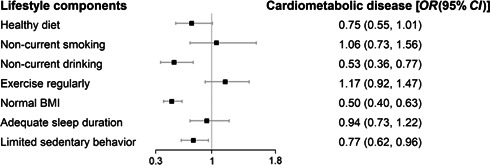
Association between healthy lifestyle components and cardiometabolic diseases. Models were adjusted for age, sex, marital status (married/other), education level (high school and below, college, above), income (low, medium, high), residence (rural, urban), birthplace (rural, urban), living alone (yes, no), family history of cardiometabolic diseases (any of hypertension, diabetes, myocardial infarction, and stroke), and mutually adjusted for each lifestyle factor. BMI, body mass index.

Among the seven lifestyle factors, normal BMI, noncurrent drinking, and limited sedentary behavior demonstrated independent associations with CMD. The *OR*s (95% *CI*s) were 0.50 (0.40–0.63) for normal BMI, 0.53 (0.36–0.77) for noncurrent drinking, and 0.77 (0.62–0.96) for limited sedentary behavior. The associations were marginally significant for healthy diet (*OR* = 0.75, 95% *CI* = 0.55–1.01) but were nonsignificant for noncurrent smoking (*OR* = 1.06, 95% *CI* = 0.73–1.56), regular exercise (*OR* = 1.17, 95% *CI* = 0.92–1.47), and adequate sleep duration (*OR* = 0.94, 95 *CI* = 0.73–1.22) (Figure 2 and Supporting Information: eTable 7).

When we examined the associations of healthy lifestyle with individual CMDs, significant beneficial associations were observed for hyperlipidemia (*OR*_T3 vs. T1_ = 0.41, 95% *CI* = 0.30–0.56) and hypertension (*OR*_T3 vs. T1_ = 0.65, 95% *CI* = 0.44–0.97) whereas no significant association was observed for diabetes (*OR*_T3 vs. T1_ = 0.93, 95% *CI* = 0.50–1.75) (Supporting Information: eTable 8). In the sensitivity analyses (Supporting Information: eTable 9), the results remained consistent when we additionally adjusted the models for depressive symptoms and anxiety symptoms, redefined healthy diet as adherence to the CHEI, redefined adequate sleep duration as 6–8 h/day, modified the lifestyle score by excluding the components of sleep duration and sedentary behavior, and used a generalized linear mixed‐effects model to account for the potential cluster effect of school.

## 4 DISCUSSION

In this cross‐sectional study among schoolteachers in eastern China, we observed that a higher overall healthy lifestyle score was significantly associated with lower odds of CMD. Compared with those who had 0–3 low‐risk factors, participants with 4 and 5–7 low‐risk factors had 34% and 49% lower odds of prevalent CMD, respectively. Normal BMI, noncurrent drinking, and limited sedentary behavior were independent contributors to the observed associations. Moreover, the associations remained consistent across major subgroups.

Our results were generally consistent with evidence from previous studies investigating the associations between adherence to a healthy lifestyle and CMD. A recent prospective study among 0.3 million participants in seven European countries found that a healthy lifestyle index (summarized with five pre‐diagnostic lifestyle behaviors including normal BMI, nonsmoking, limited alcohol consumption, and regular physical activity) was inversely associated with CVD (hazard ratio [*HR*] = 0.84, 95% *CI* = 0.79–0.90) and diabetes (*HR* = 0.82, 95% *CI* = 0.77–0.88) [1]. Another large‐scale cohort study of 0.5 million Chinese adults also found that a high‐risk lifestyle featuring unhealthy body shape, smoking, excessive alcohol consumption, physical inactivity, and poor diet was related to a higher risk of transition from healthy status to CMD (*HR* = 1.21, 95% *CI* = 1.19–1.23) and from a single CMD to cardiometabolic multimorbidity (*HR* = 1.12, 95% *CI* = 1.10, 1.15) [6]. Our study extends the evidence regarding the potentially beneficial association between an overall healthier lifestyle and CMD in the educational workforce.

In the analysis of individual lifestyle factors, we observed independent beneficial associations for normal BMI, noncurrent drinking, and limited sedentary behavior but not for noncurrent smoking, regular exercise, and healthy sleep duration. Moreover, a healthy diet showed a marginally significant association. A previous cohort study of 5476 older adults in the United Kingdom suggested that smoking, drinking, inadequate fruit and vegetable consumption, physical inactivity, and obesity were associated with a higher risk of incident cardiometabolic multimorbidity [19]. A recent cross‐sectional study conducted among 36,000 Japanese adults showed that longer sedentary time was associated with higher odds of CMD, especially dyslipidemia (*OR* = 1.12, 95% *CI* = 1.06–1.19 for younger men aged 35–49 years; *OR* = 1.10, 95% *CI* = 1.03–1.18 for women) [20]. Another meta‐analysis reported that short sleep duration (≤5–6 h/night) and long sleep duration (>8–9 h/night) were both associated with a greater risk of developing CMD [21]. Additionally, a recent large‐scale cohort of Chinese multi‐ethnic participants aged over 30 years found that greater adherence to the DASH diet was significantly associated with lower cardiometabolic risks, particularly hypertension (*OR* = 0.74, 95% *CI* = 0.70–0.79) [10]. Our findings are largely consistent with those of previous studies but also go beyond suggesting that an overall healthy lifestyle has protective associations against CMD among Chinese schoolteachers, and this association may not be limited to a single lifestyle factor.

During the ongoing COVID‐19 pandemic, social isolation has drastically affected individuals’ lifestyles [22], increasing the proportions of people with an unhealthy diet, sedentary behavior, and decreased physical activity. A recent study also reported that teachers, who had potential exposure to high‐risk situations during the pandemic, had decreased physical activities, and increased sleep problems, smoking, and drinking behaviors [23]. The subsequent unhealthy lifestyle may increase a wide range of negative cardiometabolic effects [24]. Considering that teachers are under work‐related stress and potentially at risk of developing CMDs, further studies are warranted to provide scientific evidence on lifestyle intervention and develop lifestyle guidelines for health promotion among teachers.

Our study is one of the few to evaluate lifestyle factors and CMD among schoolteachers. The careful management of potential confounding factors and design of sensitivity analyses also added to the reliability of our study findings. However, the results should be interpreted in the context of the following limitations. First, owing to the cross‐sectional study design, we could not provide evidence on the temporal relationship between lifestyle and CMD, and potential reverse causation might exist. Second, information on the lifestyle and history of CMD was self‐reported, which might be subject to potential recall bias. Nevertheless, the higher Kappa values between cardiometabolic biomarkers in health examination reports and self‐reported health status provided indirect evidence of the validity of the self‐reported measurements. Third, although our analysis controlled for major risk factors of CMD, we cannot rule out the possibility of residual and unmeasured confounding. Finally, our findings are restricted to the primary and middle school teachers in Zhejiang, China, which may limit the generalizability of the findings to other populations.

## 5 CONCLUSION

The study findings support the fact that adherence to an overall healthy lifestyle was associated with lower odds of CMD among Chinese primary and middle school teachers. Future large scale and prospective studies among schoolteachers are needed to confirm the study results.

## AUTHOR CONTRIBUTIONS

Changzheng Yuan, Ronghua Zhang, Liyan Huang, and Mengjie He designed the study; Liyan Huang performed the statistical analysis and interpreted the data; Mengjie He provided statistical support; Liyan Huang and Mengjie He developed the draft of the manuscript. Jie Shen, Yiying Gong, Hui Chen, Xiaolin Xu, Geng Zong, Yan Zheng, Chao Jiang, and Baohong Wang critically revised the manuscript for important intellectual content. Changzheng Yuan had primary responsibility for the final content. All authors have read and approved the final manuscript.

## CONFLICT OF INTEREST STATEMENT

The authors declare no conflicts of interest.

## ETHICS STATEMENT

The study protocol was approved by the medical ethics committee of the School of Public Health, Zhejiang University (ZJL202203‐4).

## INFORMED CONSENT

All participants provided electronic informed consent.

## Supporting information

Supporting information.

## Data Availability

The data are not publicly available due to privacy or ethical restrictions.

## References

[1] Freisling H , Viallon V , Lennon H , Bagnardi V , Ricci C , Butterworth AS , et al. Lifestyle factors and risk of multimorbidity of cancer and cardiometabolic diseases: a multinational cohort study. BMC Med. 2020;18(1):5. 10.1186/s12916-019-1474-7 31918762 PMC6953215

[2] Osborn LJ , Claesen J , Brown JM . Microbial flavonoid metabolism: a cardiometabolic disease perspective. Annu Rev Nutr. 2021;41:433–54. 10.1146/annurev-nutr-120420-030424 34633856 PMC12462695

[3] Shi L , Morrison JA , Wiecha J , Horton M , Hayman LL . Healthy lifestyle factors associated with reduced cardiometabolic risk. Br J Nutr. 2011;105(5):747–54. 10.1017/s0007114510004307 21276278

[4] Wijesuriya M , Fountoulakis N , Guess N , Banneheka S , Vasantharajah L , Gulliford M , et al. A pragmatic lifestyle modification programme reduces the incidence of predictors of cardio‐metabolic disease and dysglycaemia in a young healthy urban South Asian population: a randomised controlled trial. BMC Med. 2017;15(1):146. 10.1186/s12916-017-0905-6 28851373 PMC5576225

[5] Xu C , Cao Z . Cardiometabolic diseases, total mortality, and benefits of adherence to a healthy lifestyle: a 13‐year prospective UK Biobank study. J Transl Med. 2022;20(1):234. 10.1186/s12967-022-03439-y 35590361 PMC9118619

[6] Han Y , Hu Y , Yu C , Guo Y , Pei P , Yang L , et al. Lifestyle, cardiometabolic disease, and multimorbidity in a prospective Chinese study. Eur Heart J. 2021;42(34):3374–84. 10.1093/eurheartj/ehab413 34333624 PMC8423468

[7] Han H , Cao Y , Feng C , Zheng Y , Dhana K , Zhu S , et al. Association of a healthy lifestyle with all‐cause and cause‐specific mortality among individuals with type 2 diabetes: a prospective study in UK Biobank. Diabetes Care. 2022;45(2):319–29. 10.2337/dc21-1512 34857534

[8] Lloyd‐Jones DM , Allen NB , Anderson CAM , Black T , Brewer LC , Foraker RE , et al. Life's essential 8: updating and enhancing the American Heart Association's construct of cardiovascular health: a Presidential Advisory from the American Heart Association. Circulation. 2022;146(5):e18–43. 10.1161/cir.0000000000001078 35766027 PMC10503546

[9] Gao J . Association of dietary patterns and physical activities with total body fat proportions and metabolic syndrome among middle‐aged and elderly people: a cross‐sectional study. Shanghai: Fudan University; 2012.

[10] Xiao X , Qin Z , Lv X , Dai Y , Ciren Z , Yangla Y , et al. Dietary patterns and cardiometabolic risks in diverse less‐developed ethnic minority regions: results from the China Multi‐Ethnic Cohort (CMEC) Study. Lancet Reg Health West Pac. 2021;15:100252. 10.1016/j.lanwpc.2021.100252 34528018 PMC8383007

[11] Bull FC , Al‐Ansari SS , Biddle S , Borodulin K , Buman MP , Cardon G , et al. World Health Organization 2020 guidelines on physical activity and sedentary behaviour. Br J Sports Med. 2020;54(24):1451–62. 10.1136/bjsports-2020-102955 33239350 PMC7719906

[12] Nocon M , Hiemann T , Müller‐Riemenschneider F , Thalau F , Roll S , Willich SN . Association of physical activity with all‐cause and cardiovascular mortality: a systematic review and meta‐analysis. Eur J Cardiovasc Prev Rehabil. 2008;15(3):239–46. 10.1097/HJR.0b013e3282f55e09 18525377

[13] WHO Expert Consultation . Appropriate body‐mass index for Asian populations and its implications for policy and intervention strategies. Lancet. 2004;363(9403):157–63. 10.1016/S0140-6736(03)15268-3 14726171

[14] Chinese Elderly Type Diabetes Prevention and Treatment of Clinical Guidelines Writing Group, Geriatric Endocrinology and Metabolism Branch of Chinese Geriatric Society, Geriatric Endocrinology and Metabolism Branch of Chinese Geriatric Health Care Society, Geriatric Professional Committee of Beijing Medical Award Foundation, National Clinical Medical Research Center for Geriatric Diseases (PLA General) . Clinical guidelines for prevention and treatment of type 2 diabetes mellitus in the elderly in China (2022 edition). Zhonghua Nei Ke Za Zhi. 2022;61(1):12–50. 10.3760/cma.j.cn112138-20211027-00751 34979769

[15] Bureau of Disease Prevention and Control, National Health Commission of People's Republic of China, National Center for Cardiovascular Diseases, Chinese Academy of Medical Science & Peking Union Medical College, Fuwai Hospital, Chinese Center for Control and Prevention, Chinese Society of Cardiology, Chinese Medical Doctor Association Hypertension Committee, China Sport Science Society; Chinese Nutrition Society, Chinese Stroke Association, et al. National guideline for hypertension management in China (2019). Zhonghua Xin Xue Guan Bing Za Zhi. 2020;48(1):10–46. 10.3760/cma.j.issn.0253-3758.2020.01.004 32008294

[16] Joint Committee on the Chinese Guidelines for Lipid Management . 2016 Chinese guideline for the management of dyslipidemia in adults. Zhonghua Xin Xue Guan Bing Za Zhi. 2016;44(10):833–53. 10.3760/cma.j.issn.0253-3758.2016.10.005 27903370

[17] Cheng ST , Chan ACM . The Center for Epidemiologic Studies Depression Scale in older Chinese: thresholds for long and short forms. Int J Geriatr Psychiatry. 2005;20(5):465–70. 10.1002/gps.1314 15852439

[18] Spitzer RL , Kroenke K , Williams JBW , Löwe B . A brief measure for assessing generalized anxiety disorder: the GAD‐7. Arch Intern Med. 2006;166(10):1092–7. 10.1001/archinte.166.10.1092 16717171

[19] Dhalwani NN , Zaccardi F , O'Donovan G , Carter P , Hamer M , Yates T , et al. Association between lifestyle factors and the incidence of multimorbidity in an older English population. J Gerontol A Biol Sci Med Sci. 2016;72(4):528–34. 10.1093/gerona/glw146 27470302

[20] Koyama T , Kuriyama N , Ozaki E , Tomida S , Uehara R , Nishida Y , et al. Sedentary time is associated with cardiometabolic diseases in a large Japanese population: a cross‐sectional study. J Atheroscler Thromb. 2020;27(10):1097–107. 10.5551/jat.54320 32269208 PMC7585914

[21] St‐Onge MP , Grandner MA , Brown D , Conroy MB , Jean‐Louis G , Coons M , et al. Sleep duration and quality: impact on lifestyle behaviors and cardiometabolic health: a scientific statement from the American Heart Association. Circulation. 2016;134(18):e367–86. 10.1161/cir.0000000000000444 27647451 PMC5567876

[22] Balanzá–Martínez V , Atienza‐Carbonell B , Kapczinski F , De Boni RB . Lifestyle behaviours during the COVID‐19— time to connect. Acta Psychiatr Scand. 2020;141(5):399–400. 10.1111/acps.13177 32324252 PMC7264786

[23] Suze Souza e Silva N , Cabral Barbosa RE , Lemos Leão L , das Graças Pena G , de Pinho L , Almeida de Magalhães T , et al. Working conditions, lifestyle and mental health of Brazilian public‐school teachers during the COVID‐19 pandemic. Psychiatriki. 2021;32(4):282–9. 10.22365/jpsych.2021.045 34860687

[24] Bhatnagar D . The COVID‐19 pandemic: lifestyle and cardiovascular risk factors. Curr Opin Lipidol. 2021;32(1):71–3. 10.1097/mol.0000000000000725 33315619

