# Supplementary material for: Healthy lifestyles in relation to cardiometabolic diseases among schoolteachers: A cross‐sectional study
Source: Health Care Sci. 2023 Jul 10;2(4):223–32. doi: 10.1002/hcs2.59 (PMC11080911; doi:10.1002/hcs2.59)
Supplement: Supplementary file 1 — Supporting information. [file HCS2-2-223-s001.docx]

**Legends of Figures and Tables**

**eTable 1** Construction and score of Dietary Approaches to Stop Hypertension (DASH) diet

**eTable 2** Definition of healthy lifestyle factors

**eTable 3** Distributions of lifestyle factors

**eTable 4** Agreement of cardiometabolic diseases defined by cardiometabolic biomarkers and self-reported health status

**eTable 5** Spearman correlation coefficients among lifestyle factors, sociodemographic and health-related variables

**eTable 6** Performance of models evaluating the association between healthy lifestyle score and cardiometabolic diseases

**eTable 7** Subgroup analysis for the association between healthy lifestyle score and cardiometabolic diseases

**eTable 8** The associations of healthy lifestyle scores with hyperlipidemia, hypertension and diabetes

**eTable 9** Sensitivity analysis for the association between healthy lifestyle score and cardiometabolic diseases

**eFigure 10** Overview of the ongoing data collection from the Teachers ageing project (TAP)

**eTable 1** Construction and score of Dietary Approaches to Stop Hypertension (DASH) diet

| Food groups | 1 | 2 | 3 | 4 | 5 |
| --- | --- | --- | --- | --- | --- |
| Fruits | Quintile 1 | Quintile 2 | Quintile 3 | Quintile 4 | Quintile 5 |
| Vegetables | Quintile 1 | Quintile 2 | Quintile 3 | Quintile 4 | Quintile 5 |
| Nuts and legumes | Quintile 1 | Quintile 2 | Quintile 3 | Quintile 4 | Quintile 5 |
| Dairy | Quintile 1 | Quintile 2 | Quintile 3 | Quintile 4 | Quintile 5 |
| Whole grain | Quintile 1 | Quintile 2 | Quintile 3 | Quintile 4 | Quintile 5 |
| Sodium | Prefer salty taste | - | - | - | Not prefer salty taste |
| Sugar-sweetened beverages | Quintile 5 | Quintile 4 | Quintile 3 | Quintile 2 | Quintile 1 |

**eTable 2** Definition of healthy lifestyle factors

| Lifestyle | Low risk (score = 1) | High risk (score = 0) |
| --- | --- | --- |
| Diet | Higher adherence to DASH (In the top quintile of the study participants) | Low adherence to DASH (In the lower four quintiles of the study participants) |
| Physical activity | Regular exercise | Non-regular exercise |
| Smoking status | Non-current smoking (never smoking and former smoking) | Current smoking |
| Drinking status | Non-current alcohol drinking (never drinking and former drinking) | Current drinking |
| Body weight | Normal BMI (18.5-24.0 kg/m^2^) | BMI < 18.5 kg/m2;  BMI >=24.0 kg/m2 |
| Sleep duration | Adequate sleep duration (7-9 hours) | Short sleep duration (<7 hours),  Long sleep duration (>9 hours) |
| Sedentary behavior | Limited sedentary behavior (<4 hours) | More sedentary behavior (≥4 hours) |

BMI, body mass index; DASH, Dietary Approaches to Stop Hypertension

| **eTable 3** Distributions of lifestyle factors | | | | |  |  |  |
| --- | --- | --- | --- | --- | --- | --- | --- |
|  | Total | Gender | |  | Age | |  |
| Variables |  | Male | Female | P value | <50 | ≥50 | *p* value |
| Number | 2983 | 782 | 2201 |  | 2477 | 506 |  |
| DASH, *mean ± SD*, score | 26.5 (4.6) | 25.0 (4.3) | 27.0 (4.7) | <0.001 | 26.4 (4.6) | 27.0 (4.6) | 0.005 |
| <23 | 550 (19.9) | 203 (27.6) | 347 (17.1) | <0.001 | 471 (20.5) | 79 (16.8) | 0.136 |
| 23-24 | 362 (13.1) | 123 (16.7) | 239 (11.8) |  | 305 (13.3) | 57 (12.2) |  |
| 25-27 | 673 (24.3) | 191 (26.0) | 482 (23.7) |  | 564 (24.6) | 109 (23.2) |  |
| 28-30 | 613 (22.2) | 150 (20.4) | 463 (22.8) |  | 500 (21.8) | 113 (24.1) |  |
| >30 | 567 (20.5) | 68 (9.3) | 499 (24.6) |  | 456 (19.9) | 111 (23.7) |  |
| Smoking status, *n* (%) |  |  |  |  |  |  | <0.001 |
| Current | 210 (7.0) | 209 (26.7) | 1 (0.0) | <0.001 | 130 (5.2) | 80 (15.8) |  |
| Ever | 72 (2.4) | 70 (9.0) | 2 (0.1) |  | 28 (1.1) | 44 (8.7) |  |
| Never | 2701 (90.5) | 503 (64.3) | 2198 (99.9) |  | 2319 (93.6) | 382 (75.5) |  |
| Drinking status, *n* (%) |  |  |  |  |  |  | <0.001 |
| Current | 186 (6.2) | 158 (20.2) | 28 (1.3) | <0.001 | 114 (4.6) | 72 (14.2) |  |
| Ever | 309 (10.4) | 219 (28.0) | 90 (4.1) |  | 194 (7.8) | 115 (22.7) |  |
| Never | 2488 (83.4) | 405 (51.8) | 2083 (94.6) |  | 2169 (87.6) | 319 (63.0) |  |
| Exercise regularly, *n* (%) |  |  |  |  |  |  | <0.001 |
| Yes | 1135 (38.0) | 421 (53.8) | 714 (32.4) |  | 815 (32.9) | 320 (63.2) |  |
| No | 1848 (62.0) | 361 (46.2) | 1487 (67.6) | <0.001 | 1662 (67.1) | 186 (36.8) |  |
| BMI, *mean ± SD*, kg/m^2^ | 22.4 (3.3) | 24.1 (2.6) | 21.8 (3.4) | <0.001 | 22.2 (3.4) | 23.4 (2.7) | <0.001 |
| <18.5 | 235 (7.9) | 9 (1.2) | 226 (10.3) | <0.001 | 226 (9.1) | 9 (1.8) | <0.001 |
| 18.5-23.9 | 1989 (66.7) | 378 (48.3) | 1611 (73.2) |  | 1691 (68.3) | 298 (58.9) |  |
| 24.0-27.9 | 617 (20.7) | 337 (43.1) | 280 (12.7) |  | 438 (17.7) | 179 (35.4) |  |
| ≥28.0 | 142 (4.8) | 58 (7.4) | 84 (3.8) |  | 122 (4.9) | 20 (4.0) |  |
| Sleep duration, *mean ± SD*, h | 7.7 (1.5) | 7.7 (1.8) | 7.6 (1.4) | 0.221 | 7.7 (1.5) | 7.6 (1.2) | 0.264 |
| ≤6 | 558 (18.7) | 159 (20.3) | 399 (18.1) | 0.051 | 460 (18.6) | 98 (19.4) | 0.561 |
| 6-8 | 2313 (77.5) | 585 (74.8) | 1728 (78.5) |  | 1920 (77.5) | 393 (77.7) |  |
| >8 | 112 (3.8) | 38 (4.9) | 74 (3.4) |  | 97 (3.9) | 15 (3.0) |  |
| sedentary, *mean ± SD*, h | 4.0 (2.6) | 4.1 (2.6) | 4.0 (2.7) | 0.315 | 4.0 (2.7) | 4.1 (2.3) | 0.633 |
| <4 | 1482 (49.7) | 370 (47.3) | 1112 (50.5) | 0.134 | 1240 (50.1) | 242 (47.8) | 0.386 |
| ≥4 | 1501 (50.3) | 412 (52.7) | 1089 (49.5) |  | 1237 (49.9) | 264 (52.2) |  |

BMI, body mass index; DASH, Dietary Approaches to Stop Hypertension; SD, standard deviation

**eTable 4** Agreement of cardiometabolic diseases (diabetes, hypertension, or hyperlipidemia) defined by physical examination and self-reported health status

|  | **CMDs defined by physical examination** | | | | | | | |
| --- | --- | --- | --- | --- | --- | --- | --- | --- |
|  | Type 2 diabetes (*n*=88) | |  | Hypertension  (*n*=50) | |  | Hyperlipidemia  (*n*=87) | |
|  | Yes | No |  | Yes | No |  | Yes | No |
| **Self-reported history of CMDs** | | | | | | | | |
| Yes | 3 | 0 |  | 4 | 1 |  | 11 | 1 |
| No | 2 | 83 |  | 2 | 43 |  | 27 | 48 |
| **Kappa coefficient** | 0.977 | |  | 0.940 | |  | 0.678 | |

CMD, cardiometabolic disease

| **eTable 5** Spearman correlation coefficients among lifestyle factors, sociodemographic and health-related variables | | | | | | | | | | | | | | | | | |
| --- | --- | --- | --- | --- | --- | --- | --- | --- | --- | --- | --- | --- | --- | --- | --- | --- | --- |
|  | Physical activity | Smoking | Drinking | BMI | Sleep duration | Sedentary behavior | Diet | Age | Gender | Marriage | Education level | Personal income level | Residence place | Birth place | Living alone | History of CMD | Family history of CMD |
| Physical activity | 1 |  |  |  |  |  |  |  |  |  |  |  |  |  |  |  |  |
| Smoking | -0.10 | 1 |  |  |  |  |  |  |  |  |  |  |  |  |  |  |  |
| Drinking | -0.09 | 0.34 | 1 |  |  |  |  |  |  |  |  |  |  |  |  |  |  |
| BMI | -0.01 | 0.16 | 0.11 | 1 |  |  |  |  |  |  |  |  |  |  |  |  |  |
| Sleep duration | 0.04 | 0.05 | 0.04 | 0.01 | 1 |  |  |  |  |  |  |  |  |  |  |  |  |
| Sedentary behavior | 0.13 | -0.01 | -0.02 | 0.02 | 0.07 | 1 |  |  |  |  |  |  |  |  |  |  |  |
| Diet | 0.12 | 0.09 | 0.04 | 0.09 | 0.05 | 0.06 | 1 |  |  |  |  |  |  |  |  |  |  |
| Age | 0.23 | -0.16 | -0.17 | -0.02 | -0.04 | 0.04 | 0.08 | 1 |  |  |  |  |  |  |  |  |  |
| Gender | -0.19 | 0.46 | 0.34 | 0.23 | 0.04 | 0.03 | 0.16 | -0.20 | 1 |  |  |  |  |  |  |  |  |
| Marriage | 0.03 | -0.04 | -0.06 | 0.03 | 0.04 | 0.09 | 0.06 | 0.46 | -0.03 | 1 |  |  |  |  |  |  |  |
| Education level | -0.05 | 0.07 | 0.05 | 0.01 | 0.02 | -0.07 | 0.04 | -0.21 | 0.07 | -0.09 | 1 |  |  |  |  |  |  |
| Personal income level | 0.05 | -0.04 | -0.05 | 0.01 | 0.01 | 0.01 | 0.08 | 0.20 | -0.06 | 0.15 | 0.08 | 1 |  |  |  |  |  |
| Residence place | -0.05 | -0.02 | -0.02 | -0.07 | 0.01 | -0.03 | -0.05 | -0.14 | -0.05 | -0.17 | -0.07 | -0.12 | 1 |  |  |  |  |
| Birth place | 0.01 | -0.07 | -0.06 | -0.03 | 0.00 | -0.01 | -0.05 | 0.07 | -0.13 | 0.03 | -0.05 | -0.02 | 0.23 | 1 |  |  |  |
| Living alone | 0.00 | 0.00 | -0.03 | 0.01 | 0.03 | 0.06 | 0.07 | 0.26 | 0.01 | 0.50 | -0.15 | 0.05 | 0.04 | 0.01 | 1 |  |  |
| History of CMD | 0.11 | -0.15 | -0.20 | -0.16 | -0.03 | -0.04 | -0.05 | 0.32 | -0.24 | 0.12 | -0.09 | 0.05 | 0.00 | 0.03 | 0.06 | 1 |  |
| Family history of CMD | 0.01 | -0.01 | -0.05 | -0.01 | -0.05 | 0.00 | 0.01 | 0.23 | -0.01 | 0.13 | -0.06 | 0.05 | -0.06 | -0.01 | 0.06 | 0.20 | 1 |

BMI, body mass index; CMD, cardiometabolic disease

| **eTable 6** Performance of models evaluating the association between healthy lifestyle score and cardiometabolic diseases | | | | | | | |
| --- | --- | --- | --- | --- | --- | --- | --- |
|  | OR (95%CI) for lifestyle score tertiles | | |  | Performance of models | | |
|  | Low | Medium | High |  | AIC | BIC | R^2^ |
| Model 1 | Ref | 0.66 (0.50, 0.87) | 0.79 (0.72, 0.86) |  | 2251 | 2293 | 0.16 |
| Model 2 | Ref | 0.66 (0.50, 0.86) | 0.79 (0.72, 0.86) |  | 2258 | 2348 | 0.16 |
| Model 3 | Ref | 0.66 (0.50, 0.88) | 0.80 (0.73, 0.88) |  | 2199 | 2294 | 0.19 |

AIC, Akaike information criterion; BIC, Bayesian information criterion; OR, odds ratio; CI, confidence interval

Model 1 adjusted for age (<30/ 30-39/ 40-49/ ≥50), gender;

Model 2 additionally adjusted for married status (Married/ other), education level (high school and below/ bachelor /masters or doctors), income (low/ medium/ high), residence (rural/ urban), birthplace (rural/ urban), and living alone (yes/ no);

Model 3 additionally adjusted for family history of cardiometabolic diseases (any of hypertension, diabetes, and myocardial infarction).

| **eTable 7** Subgroup analysis for the association between healthy lifestyle score and cardiometabolic diseases | | | | |
| --- | --- | --- | --- | --- |
|  | Low risk vs High risk | | |  |
|  | Model 1 | Model 2 | Model 3 | Model 4 |
| Lifestyle components |  |  |  |  |
| Healthy diet | 0.73 (0.54, 0.97) | 0.74 (0.55, 0.98) | 0.73 (0.54, 0.97) | 0.75 (0.55, 1.01) |
| Non-current smoking | 0.91 (0.64, 1.31) | 0.91 (0.64, 1.31) | 0.88 (0.61, 1.27) | 1.06 (0.73, 1.56) |
| Non-current drinking | 0.50 (0.35, 0.72) | 0.50 (0.35, 0.72) | 0.53 (0.37, 0.76) | 0.53 (0.36, 0.77) |
| Exercise regularly | 1.00 (0.80, 1.25) | 1.02 (0.82, 1.27) | 1.06 (0.85, 1.33) | 1.17 (0.92, 1.47) |
| Normal BMI | 0.50 (0.40, 0.62) | 0.49 (0.39, 0.61) | 0.50 (0.40, 0.62) | 0.50 (0.40, 0.63) |
| Normal sleep duration | 0.88 (0.69, 1.13) | 0.87 (0.68, 1.11) | 0.91 (0.71, 1.16) | 0.94 (0.73, 1.22) |
| Short sedentary duration | 0.80 (0.65, 0.99) | 0.79 (0.64, 0.98) | 0.80 (0.64, 0.99) | 0.77 (0.62, 0.96) |
| Model 1 adjusted for age, gender | | | | |
| Model 2 additionally adjusted for age, gender, marriage (Married/ other), education level (high school and below/ bachelor /masters or doctors), income (<10/ 10-20/ >20), residence place (rural/ urban), birth place (rural/ urban), resident (alone/ with others) | | | | |
| Model 3 additionally adjusted for family history of cardiometabolic diseases (any of hypertension, diabetes, and myocardial infarction). | | | | |
| Model 4 additionally adjusted for each healthy lifestyle factor mutually | | | | |
| ^*^ *p*<0.05, ^**^ *p*<0.01, ^***^ *p*<0.001 | | | | |

**eTable 8** The associations of healthy lifestyle scores with hyperlipidemia, hypertension and diabetes

| Variables | Lifestyle | | | Continuous | *P*-trend |
| --- | --- | --- | --- | --- | --- |
|  | Low | Medium | High |  |  |
| Score range | 0-3 | 4 | 5-7 | 0-7 |  |
| *N* | 657 | 980 | 1346 | 2983 |  |
| Hyperlipidemia | Ref | 0.70 (0.52, 0.94) | 0.41 (0.30, 0.56) | 0.74 (0.66, 0.81) | <0.001 |
| Hypertension | Ref | 0.58 (0.38, 0.87) | 0.65 (0.44, 0.97) | 0.87 (0.77, 0.99) | 0.036 |
| Diabetes | Ref | 0.87 (0.45, 1.66) | 0.93 (0.50, 1.75) | 1.01 (0.83, 1.23) | 0.934 |

Models were adjusted for age, gender, marriage (Married/ other), education level (high school and below/ bachelor /masters or doctors), income (<10/ 10-20/ >20), residence place (rural/ urban), birth place (rural/ urban), resident (alone/ with others) and family history of cardiometabolic diseases (any of hypertension, diabetes and myocardial infarction).

**eTable** **9** Sensitivity analysis for the association between healthy lifestyle score and cardiometabolic diseases

| Variables | Lifestyle | | | Continuous | *p*-trend |
| --- | --- | --- | --- | --- | --- |
|  | Low | Medium | High | (per 1 unit) |  |
| **Sensitivity analysis 1**: Additionally adjusted for depressive symptoms and anxiety symptoms | | | | | |
| Score range | 0-3 | 4 | 5-7 | 0-7 |  |
| *N* | 657 | 980 | 1346 | 2983 |  |
| OR (95%CI) ^a^ | Ref | 0.69 (0.52, 0.91) | 0.55 (0.41, 0.72) | 0.82 (0.75, 0.90) | <0.001 |
| **Sensitivity analysis 2**: Redefined healthy diet as highest adherence to Chinese Healthy Eating Index | | | | | |
| Score range | 0-3 | 4 | 5-7 | 0-7 |  |
| *N* | 641 | 975 | 1367 | 2983 |  |
| OR (95%CI) ^b^ | Ref | 0.68 (0.51, 0.90) | 0.52 (0.39, 0.69) | 0.81 (0.74, 0.88) | <0.001 |
| **Sensitivity analysis 3**: Redefined normal sleep duration as 6-8 hours | | | | | |
| Score range | 0-3 | 4 | 5-7 | 0-7 |  |
| *N* | 619 | 960 | 1404 | 2983 |  |
| OR (95%CI) ^b^ | Ref | 0.66 (0.50, 0.87) | 0.48 (0.36, 0.64) | 0.78 (0.71, 0.86) | <0.001 |
| **Sensitivity analysis 4**: Redefined lifestyle score as the summarization of five modifiable lifestyle factors, including diet, smoking, drinking, physical activity and body mass index | | | | | |
| Score range | 0-2 | 3 | 4-5 | 0-5 |  |
| *N* | 625 | 1029 | 1329 | 2983 |  |
| OR (95%CI) ^b^ | Ref | 0.59 (0.46, 0.77) | 0.45 (0.33, 0.60) | 0.75 (0.67, 0.84) | <0.001 |
| **Sensitivity analysis 5**: Used generalized linear mixed effect models with school as random effect | | | | | |
| Score range | 0-3 | 4 | 5-7 | 0-7 |  |
| *N* | 657 | 980 | 1346 | 2983 |  |
| OR (95%CI) ^b^ | Ref | 0.66 (0.50, 0.87) | 0.50 (0.38, 0.66) | 0.80 (0.73, 0.87) | <0.001 |

^a^ Models adjusted for age, gender, marriage (Married/ other), education level (high school and below/ bachelor /masters or doctors), income (<10/ 10-20/ >20), residence place (rural/ urban), birth place (rural/ urban), resident (alone/ with others), depressive symptoms and anxiety symptoms. ^b^ Models adjusted for age, gender, marriage (Married/ other), education level (high school and below/ bachelor /masters or doctors), income (<10/ 10-20/ >20), residence place (rural/ urban), birth place (rural/ urban), resident (alone/ with others) and family history of cardiometabolic diseases (any of hypertension, diabetes and myocardial infarction).
